# Addressing Oral Health Disparities of a Tribal Population Through a Combined Implementation of Focus Group Discussion, Mobile Technology Networking, and Creating a Supportive Environment: A Prospective Study

**DOI:** 10.7759/cureus.41266

**Published:** 2023-07-01

**Authors:** Minti Kumari, Swati Sharma, Anil Raj, Ankita Jha, Sahana Shivakumar, Alok kumar

**Affiliations:** 1 Public Health Dentistry, Patna Dental College and Hospital, Patna, IND; 2 Pedodontics and Preventive Dentistry, Dental College, Rajendra Institute of Medical Sciences (RIMS), Ranchi, IND; 3 Periodontics, Patna Dental College and Hospital, Patna, IND; 4 Public Health Dentistry, People's College of Dental Sciences and Research Centre, Bhopal, IND; 5 Oral Medicine and Radiology, Private Practice, Patna, IND

**Keywords:** oral health, gingival health, oral health care, oral health promotion, focus group discussion, mobile networking

## Abstract

Background and objective: Oral health disparities generally exist among tribal populations, prompting creative solutions to tackle these challenges. By using a combined implementation strategy of including focus group discussion (FGD), mobile technology networking (MTN), and creating a supportive environment, this study aims to assess and bring positive changes in oral health in these populations.

Methods: The current study employed a mixed-method approach on a sample of 100 tribal volunteers. Qualitative assessment included FGD conducted regularly for three months based on themes such as oral hygiene habits, access to oral health, technology in oral health, the relationship of oral health to general health, and the role of diet in oral health. Quantitative evaluation included recording of the oral hygiene index-simplified and gingival index to measure gingival status. Messages on oral health were routinely posted to mobile phones to reinforce oral health education. Appropriate use of indigenous oral hygiene aids (neem and datun) was also taught during the discussion session. Clinical examinations were compared before and after FGD. Data were analyzed using IBM SPSS Statistics for Windows, Version 25 (Released 2017; IBM Corp., Armonk, New York, United States). A paired ‘t’ test was used to find significant differences in gingival status at p<0.05.

Results: The FGD sessions deduced observations such as limited access to dental care, inadequate oral hygiene practices such as usage of neem sticks and twigs, and lack of oral health awareness. The implementation of MTN facilitated the dissemination of oral health information and enhanced communication between community members and healthcare providers. The gingival index score significantly improved from pre-FGD to post-FGD with a mean difference of 0.41700 significant at p=0.000. Oral hygiene of the target population shifted from “Fair” oral hygiene status to “Good” oral hygiene status.

Conclusion: The combined implementation of FGD, MTN, and creation of a supportive environment demonstrated promising results in addressing oral health disparities among the tribal population. The interventions led to improved gingival status and better utilization of oral hygiene practices. These findings highlight the importance of tailored interventions, community engagement, and mobile technology in addressing oral health disparities in tribal populations. Ongoing support, sustainability, and further research are necessary to ensure the long-term impact and effectiveness of these interventions.

## Introduction

Oral health disparities are a significant public health concern, particularly among tribal populations [1]. These disparities are influenced by a range of factors, including limited access to dental care, cultural beliefs, poverty, and lack of oral health education. Addressing these disparities requires a multifaceted approach that takes into account the unique needs and circumstances of the tribal population [2]. In this context, a combined implementation of focus group discussion (FGD), mobile technology networking (MTN), and creating a supportive environment is an effective strategy that can improve oral health outcomes among the tribal population. By identifying the population and their oral health needs, developing an oral health promotion program, implementing the program through a community-based approach, and creating a supportive environment, this strategy can help participants adopt healthy oral hygiene habits and adhere to oral health recommendations [3]. This approach has the potential to improve the oral health of tribal populations and reduce the oral health disparities that exist within these communities [4]. Regular evaluation of the program is also essential to ensure its effectiveness and sustainability.

Literature has data on the oral health status of various tribal communities in India [5-7]. But none exist on the Gond tribes in the Raisin district area. These represent a sect who are isolated from the hustles of city life. Health education interaction done at a single point and by a Socratic approach cannot effectively retain the learned behavior. FGD is a form of research methodology that brings together a group of people to respond to questions in a moderated environment. It will give them a chance to put forth their views and problems and help bring in suitable solutions. This form of discussion is totally different from that of mass education, wherein the health educator only speaks and there is minimal or literally no interaction at all. Mobile phones are extensively used even in remote areas of community dwellings. Presenting short messages in mobiles on a regular basis will further reinforce what was discussed during FGD. Making “healthier choices the easier choices” is the baseline of creating supportive ecology in health. In our context, it was achieved by educating the tribals about appropriate use of indigenous oral hygiene aids. Generally, changes in oral health bought after interactive sessions are subjectively measured, which always has a hidden element of bias. This study is the first of its kind that assesses oral health changes brought about by the combined implementation objectively by reliable quantitative indices, thus eliminating any errors. Hence, the present study was undertaken to answer the research question “Is there an improvement in gingival and oral hygiene status after implementing the combined model of FGD, mobile networking, and creating a supportive environment using indigenous oral hygiene aids in a tribal population?”

## Materials and methods

The study design was a community-led trial using a convergent parallel mixed method model. Ethical clearance to conduct the study was obtained from the Institutional Ethical Committee of People's College of Dental Sciences and Research Centre, Bhopal (EC-202111). Informed consent from all 100 tribal volunteers was taken after explaining the study details. Confidentiality of every subject was maintained.

Sample size and technique

A convenient sampling technique was employed to recruit the study participants. Random recruitment was difficult in our study as we needed participants who were willing to actively participate and respond in a group. Convenience sampling is a non-probability sampling technique commonly used in pilot studies and exploratory research. The researcher selects participants based on their convenience or proximity to the research setting. A total of 100 tribal volunteers were chosen for the study. The study population was recruited from village habitats of tribes in Raisin district, Madhya Pradesh. A set of 10 FGD teams were made, with 10 in each group. The samples were conveniently chosen after checking for their eligibility criteria.

Eligibility criteria

Participants between 18 and 45 years of age of both genders who remained in their tribal habitats throughout life were recruited. The respondents were chosen only when they were willing to participate in FGDs and engage with MTN. 

Three examiners who were calibrated for codes and criteria of the indices conducted the clinical examination. The Kappa score showed an inter-examiner reliability of 0.87 and an intra-examiner reliability of 0.91 suggesting good agreement in scoring the codes.

Method of intervention 

It included both quantitative and qualitative aspects. Clinical examinations for the gingival status made up for the quantitative evaluation while FGD was categorized as the qualitative assessment. Data collection was conducted in three phases. The first phase included the evaluation of the gingival status of all included participants through gingival indices. The second phase constituted regular and systematic FGDs and mobile networking. FGD was conducted by the examiners who performed clinical evaluation. In the last phase, gingival health status was re-evaluated.

Quantitative phase or oral health status examination or first phase 

Gingival and oral hygiene status outcomes were measured using the gingival index [8] and oral hygiene index-simplified [9]. The gingival index was scored as "0 = normal gingiva", "1 = mild inflammation", "2 = moderate inflammation", and "3 = severe inflammation". Oral hygiene index-simplified was scored as "0 -1.2 = Good", "1.3 - 3.0 = Fair", and "3.1 - 6.0 = Poor". 

Qualitative phase or second phase

Participants were categorized into groups of 10. Groups were arranged to include similar gender and age ranges for better discussion. The facilitator conducted the discussion in the local language to ensure that the participants were comfortable and could express their opinions freely. FGD aimed at topics such as oral health habits, oral health education, access to dental care, community oral health, oral health, and overall, health, oral health, and technology. These questions and concepts guided a productive discussion on oral health in a focus group setting.

The themes or topics of FGD were put forth to all groups and open discussion was encouraged for a session of 45 - 60 minutes. Discussions were done regularly at monthly intervals for a period of three months. A social media platform (WhatsApp) group was also formed of all the participants. Subsequently, videos related to oral health and appropriate oral hygiene practices were shown and shared with all via mobile phones. Following the FGD session, demonstration of appropriate use of indigenous oral hygiene aids such as datun sticks and plain mouth water rinsing were taught by the authors.

The collected data were analyzed using IBM SPSS Statistics for Windows, Version 25 (Released 2017; IBM Corp., Armonk, New York, United States). Oral hygiene levels and gingivitis before and after intervention were measured by applying the paired 't'-test at p <0.05. 

## Results

All 100 study participants completed the intervention. FGD was conducted twice in the three-month span. The mean age of the participants was 36.04 + 10.11 years. A clear female predilection (77 females versus 23 males) was noted. The majority of them were into agricultural farming (87%) followed by poultry and craft work. It was observed during the study that participants were enthusiastic and keenly interacted with the facilitator.

Table 1 enlists the various sections of FGD, the questions in each section, and the themes deduced from the discussion; 93.8% of the sample felt that oral health was not related to general health suggesting poor dental health awareness. Oral hygiene practices in the family did not differ between its members. Neither technology was not thought to improve oral health nor the significance of diet was known to the study respondents. The tribal population was clueless about mental health awareness and its relation to oral health. Each theme of FGD was elaborately discussed, and the main deduction was noted down. FGD identified that oral health awareness was compromised in the tribal sect evaluated. Overall, tribals were not well aware of oral health and the factors associated with it.

**Table 1:** Themes and deductions of FGD FGD: Focus group discussion

Themes	Deductions
Oral hygiene habits	Half of the population used twigs and neem sticks to clean their teeth. A negligible portion used a toothbrush and paste.
Oral health education	Oral hygiene practices were learnt from family members in all the participants. Though mobile phones were used commonly, they did not influence their knowledge or practice of oral hygiene
Access to dental care	There was no access to dental care in their habitats. Distance and cost were the major reasons for avoidance of dental treatment.
Community oral health	The importance of oral health was not considered at par with general health by 93.8% of the tribal population.
Challenges to oral health	Elderly members complained of compromised mastication and nutrition due to edentulousness. Young adults and females were more concerned about aesthetics.
Oral health and technology	None of them thought technology could be used to improve oral health.
Oral health and diet	None in the target population were aware of the role of diet in improving oral health.
Role of mental health in oral health	Role of sound mind in maintaining their oral health was first heard from the facilitator of FGD.

Table 2 presents the findings of oral hygiene practices in tribal samples. Fifty-four percent of the population used neem sticks and datun to clean their teeth and only 21 % used toothpaste. Charcoal was also used in 13%. This suggests that oral hygiene practices of the tribals were subpar. Thirty-seven percent of the study sample brushed lesser than once daily, while a majority (56.0%) brushed once a day, thus highlighting the lack of appropriate oral hygiene practices. 

**Table 2:** Oral hygiene practices among the tribal population

Variables	Expressed as n (%)			
Materials	Paste	Powder	Neem	Charcoal
	21 (21.0 %)	12 (12.0%)	54 (54.0%)	13 (13.0%)
Frequency of cleaning	< Once daily	Once daily	Twice daily	
	37 (37.0 %)	56 (56.0 %)	7 (7.0 %)	

Table 3 demonstrates the change in gingival health before and after FGD intervention. Gingival status as measured by the gingival index significantly improved from pre-FGD to post-FGD. Gingival health also reduced to 0.7246 + 0.09524 from 1.1416 + 0.12315, with a mean difference of 0.41700, significant at p=0.000. Oral hygiene index-simplified reported a score of 1.4964 + 0.26577 that reduced to 0.9574 + 0.08994 after FGD. Oral hygiene index-simplified of the target population shifted from “Fair Oral hygiene status (ranging from 1.2 to 3.0) to “Good Oral hygiene status (Ranged 0 - 1.2). A significant improvement in gingival health was noted by the combined intervention employed.

**Table 3:** Comparison of oral hygiene and gingival scores before and after FGD *: Significant; FGD: focus group discussion

Variable	Interval	Mean	S.D	Mean Difference	‘t’ test	P value
Oral hygiene Index-simplified	Pre – FGD	1.4964	.26577	.53900	15.533	0.000*
	Post – FGD	.9574	.08994			
Gingival Index	Pre – FGD	1.1416	.12315	.41700	17.388	0.000*
	Post FGD	.7246	.09524			

## Discussion

The present study was conducted on 100 Gond tribal volunteers of the Raisin district and is the first of its kind. It was noted that 54% of the sample used neem twigs for oral hygiene maintenance suggesting decreased accessibility to oral health care products. Knowledge levels regarding dental health were also lesser with only 21% of the population using toothpaste. A definite improvement in gingival health was noted with the decrease in both oral hygiene index-simplified and gingival index scores suggesting the effectiveness of the combined intervention strategy. 

FGD aimed to understand the oral health knowledge, attitudes, beliefs, and practices of the population. FGD was facilitated in the local language to ensure that participants were comfortable and could express their opinions freely. A volunteer from their habitat during FGD helped us to easily integrate the program into the community. The use of MTN was integrated into the program to ensure that the participants received ongoing support and reminders. Practices such as appropriate use of datun sticks and mouth rinsing with water after every meal remains crucial to sustaining oral health promotion. By creating a supportive environment, participants are more likely to adopt healthy oral hygiene habits and adhere to oral health recommendations. The findings from this study might help in the development of an oral health promotion program that addresses the specific needs of the population.

Our study reported that greater than half of the population used neem sticks and twigs to clean their teeth. The study by Asif et al. reported similar findings with only 22% of Koya tribes and 30.80% of Lambada tribes using toothpaste and a good number using sand and charcoal [7]. Similar outcomes were observed in the rural population of the Gambia in West Africa by Jordan, where a large proportion of people utilized chew sticks (50.6%) and toothbrushes (34.6%) [10]. In contrast, Naheeda et al. observed that the majority of the population used a toothbrush and toothpaste and brushed their teeth once per day in their study conducted among tribals of the Khammam district [11]. Considering the economic constraints of this population, a simple toothbrush and paste were also difficult to afford by most of them. For those who were already using a toothbrush and paste, they were strongly encouraged to follow the same. The sect of the population who could absolutely not afford was taught the rightful usage of neem sticks and datun.

OHI - S ratings reduced from “Fair (1.4964 + 0.2657)” to “Good (0.95874 + 0.08994)”, which was significant at p=0.000. The positive change in oral health bought by the combined strategy can be well appreciated. As the study design in itself is novel, we could only compare our pre-FGD with existing literature. Pre-FGD scores of OHI-S were in accordance with the study by Asif et al. who also reported “Fair score” for Koya and Lambada tribes [7]. This is also in concordance with tribals residing in Telangana [12]. Kumar et al. showed 57.0% of their study respondents showed poor oral hygiene. The oral health disparities of the tribal population can be attributed to various factors such as limited access to dental services, lack of oral health education, poverty, and cultural beliefs [13].

Seeking prompt dental treatment and proper oral hygiene practices enhances oral health. Improving oral health can have a significant positive impact on oral health-related quality of life [14]. While the combined implementation is an effective strategy to address oral health disparities among tribal populations, there are some limitations to this approach. One limitation is the availability of resources to implement the program. The implementation of this approach requires significant financial, material, and human resources, which may not be available in all settings. Additionally, the program's sustainability may be affected if these resources are not sustained in the long term. Another limitation is the language barrier. The approach requires conducting the FGD and providing oral health education in the local language, which may be challenging if there is no standard written language or if the language is not widely spoken. This limitation can be overcome by employing interpreters or utilizing bilingual staff. The approach may also be limited by cultural beliefs and practices that may influence oral health behaviors. Some cultures may not prioritize oral health, or traditional practices may contradict modern oral health recommendations. Addressing these cultural beliefs and practices requires sensitivity and cultural competency on the part of the program implementers. Though the combined implementation is an effective strategy to address oral health disparities among tribal populations, it is essential to consider these limitations and tailor the program to the specific needs and circumstances of the population.

The utilization of FGD allowed us to gain in-depth insights into the oral health challenges faced by the tribal population. It provided a platform for open discussions, allowing community members to express their concerns, beliefs, and knowledge regarding oral health. The FGD sessions helped us understand the specific needs and priorities of the community. The integration of MTN played a pivotal role in enhancing access to oral health information and services. MTN has the advantage of reaching a larger population, even those in remote areas. It also provides educational materials, oral hygiene tips, and appointment reminders, empowering individuals to take charge of their oral health. In fact, it can act as a facilitated communication between healthcare providers and community members, enabling timely consultations and referrals. Creating a supportive environment can indeed be a crucial aspect of an intervention aimed at addressing oral health disparities in a tribal population. A supportive environment plays a significant role in promoting positive oral health behaviors and outcomes. 

Based on the current literature and the experience of implementing the research, several recommendations can be made for future efforts in this area.

· In order to identify service gaps and enhance the efficacy of the strategy, it is crucial to frequently examine the oral health needs of indigenous groups. Periodic surveys, focus groups, and community-based participatory research are effective ways to accomplish this.

· There is a need to expand relationships between public health organizations, tribal administrations, and academic institutions in order to increase the resources available for putting the strategy into practice.

· Further research on the use of mobile technologies is warranted, especially in terms of reaching out to tribal populations that have little access to oral health care. This can involve the use of mobile applications for oral health education and support, text message reminders, and telehealth services [15].

· In order to make oral health promotion programs more acceptable to these tribals and culturally suitable, it is necessary to incorporate traditional knowledge and practices. This can be done by carefully collaborating with local community leaders and healers to incorporate ancient practices into current oral health recommendations.

## Conclusions

Our combined approach of FGD, MTN, and creating a supportive environment yielded promising results. We observed improved gingival health among the intervened tribal population. Furthermore, there was a notable reduction in oral health disparities, as evidenced by improved oral health outcomes among the tribal population. However, it is important to acknowledge that addressing oral health disparities is an ongoing process, and there are still challenges to overcome. Sustaining the interventions and ensuring their long-term impact will require continued support from healthcare providers, policymakers, and community stakeholders. Additionally, further research is needed to evaluate the long-term effectiveness and cost-effectiveness of the interventions, as well as to explore other potential factors contributing to oral health disparities in the tribal population.
